# Cortisol predicts migration timing and success in both Atlantic salmon and sea trout kelts

**DOI:** 10.1038/s41598-019-39153-x

**Published:** 2019-02-20

**Authors:** Kim Birnie-Gauvin, Hugo Flávio, Martin L. Kristensen, Sarah Walton-Rabideau, Steven J. Cooke, William G. Willmore, Anders Koed, Kim Aarestrup

**Affiliations:** 1 DTU Aqua, National Institute of Aquatic Resources, Section for Freshwater Fisheries Ecology, Technical University of Denmark, , Vejlsøvej 39, 8600 Silkeborg, Denmark; 2 Fish Ecology and Conservation Physiology Laboratory, Department of Biology, Carleton University, 1125 Colonel By Dr, Ottawa, K1S 5B6 Canada; 3 Institute of Biochemistry, Department of Biology and Chemistry, Carleton University, 1125 Colonel By Dr, Ottawa, K1S 5B6 Canada

## Abstract

Kelts – individuals of anadromous fish species which have successfully spawned and may return to sea to repeat the cycle – are perhaps the least studied life stage of iteroparous fish species. To date, our understanding of what makes them successful in their return migration to sea is limited. We investigated the relationship between three physiological parameters (baseline cortisol, baseline glucose and low molecular weight antioxidants) and the timing and success of Atlantic salmon (*Salmo salar*) and sea trout (*Salmo trutta*) kelt migration. To do so, we combined blood samples obtained within 3 minutes of capture and acoustic telemetry to track 66 salmon and 72 sea trout as they migrated out of rivers, into fjords and out at sea. We show that baseline cortisol may be a good predictor of migration success. Individuals with high baseline cortisol levels exited the river earlier but were less likely to successfully reach the sea. Similar relationships were not observed with glucose or antioxidants. We provide the first evidence to support the role of physiological status in migration success in Atlantic salmon and sea trout kelts. Our findings contribute to our understanding of the relationship between physiology and fitness in wild animals. Further, we suggest that migration timing is a trade-off between stress and readiness to migrate.

## Introduction

Anadromous salmonids migrate from freshwater to sea as smolts to maximize their somatic growth and reproductive potential, and later return to freshwater to spawn^1^. Combined with the demands of smoltification as juveniles and reproductive maturation as adults, these transitions between two vastly different environments are demanding^2^. Physiologically, individuals must acclimate to freshwater through critical osmoregulatory processes^3,4^ whilst traveling the long journey to suitable spawning grounds, and must do so on a limited energy budget^5^.

Survival at sea and during return migrations is viewed as a substantial obstacle to future population sustainability, with many salmonid populations declining in recent years due to elevated mortality rates^6,7^. To date, most studies have either focused on the survival of smolts in rivers or as they enter marine environments^8–10^, and on the survival of adults as they make their way up to spawning grounds^11–13^. These studies have merit in informing conservation and management authorities about the current bottlenecks in the salmonid lifecycle. However, there has been less focus on the survival of kelts – individuals that have spawned and may return to sea to repeat the cycle (though see)^14,15^. Because some salmonids, such as Atlantic salmon (*Salmo salar*) and brown trout (*Salmo trutta*), can undergo spawning migrations more than once (i.e., they are iteroparous), repeat spawners may contribute greatly to population maintenance and recruitment^14,16^. Within this context, there has been little focus on the mechanisms which may affect a fish’s success in returning to sea post-spawning.

In brown trout for example, it was found that pre-spawning, but not post-spawning, migration was related to river discharge and temperature^17^, suggesting that other factors may be at play in influencing the timing and success of post-spawning migration. Perhaps, these factors are related to underlying physiological status. There is extensive evidence to support the physiological basis for spawning migration timing and success in Pacific salmon species (*Oncorhynchus* spp.). For example, studies have revealed correlations between river entry or migration success and physiological parameters including testosterone levels, plasma potassium and chloride ions, glucose, lactate, osmolality, and stress-regulating gene expression^12,18–20^.

In the present study, we investigated the potential relationship between three physiological parameters (baseline cortisol, baseline glucose and antioxidants) and kelt outmigration timing and success. Kelts are perhaps the least studied life stage of salmonids, despite their potential importance for population maintenance and recruitment. We chose to investigate baseline cortisol – the primary glucocorticoid stress hormone in fish^21,22^ – because it serves as a biomarker for stress exposure, and thus may provide insight on individual stress levels^23,24^. Baseline glucose was chosen because increased gluconeogenesis is associated with the upregulation of cortisol^23^ and thus it provides information on acute stress levels and energy status^25,26^. A measure of antioxidant capacity was also included because there is growing evidence suggesting a link between oxidative stress processes, including antioxidants, and ecological phenomena^27^. For example, it has been stipulated that oxidative stress is likely to increase during migrations due to elevated metabolic demands^28^. Existing findings support both a decrease or increase in antioxidants as a response of increased oxidative stress however^29^, making it difficult to make meaningful predictions. Nonetheless, a recent study showed that brown trout smolts with higher antioxidant capacity migrated earlier perhaps as an indication of readiness to migrate^30^. We therefore hypothesized that kelts with higher antioxidant capacity may do the same, and migrate earlier (herein defined as day of river exit). Based on previous findings, which suggest that spawning success is associated with lower cortisol and glucose levels, we postulated that fish with lower plasma cortisol and glucose were more likely to successfully return to sea. We further postulated that individuals with higher plasma cortisol and glucose would delay their migration, and exit the river later, perhaps as a way to maximize their probability of success.

## Material and Methods

### Study sites

**Figure 1:**
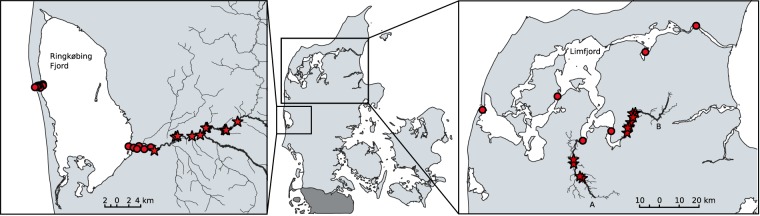
*(left)* River Skjern, Western Jutland, Denmark, where Atlantic salmon (*Salmo salar*) were tagged and released. Kelts exit the river (6 ALS) in the Ringkøbing Fjord (4 ALS on the fjord side, 4 ALS on the seaside). (*right*) Rivers Karup (A) and Simested (B), Northern Jutland, Denmark, where sea trout (*S. trutta*) were tagged and released. Kelts exit the rivers (1 ALS at each river mouth) into the Limfjord (4 ALS throughout) Red circles indicate acoustic receivers. Stars indicate release sites.

Atlantic salmon were obtained from River Skjern situated in western Jutland, Denmark, which runs for approximately 100 km, before exiting in Ringkøbing Fjord (Fig. 1). It is home to one of the largest Atlantic salmon populations in Denmark. The population is maintained both through naturally-producing individuals and supportive stocking of fish native to the river itself. Salmon kelts were caught in the lower 20 km of the river, and can only exit to the fjord through the outlet of river Skjern. Sea trout were obtained from River Karup and Simested situated in northern Jutland, Denmark. The rivers run for approximately 78 and 34 km, respectively, before exiting in the Limfjord (Fig. 1). Sea trout kelts were caught in the lower 20 km of rivers Karup and Simested, each with a single exit to the Limfjord. While the Limfjord has two exits, sea trout have only ever been detected on the eastern outlet, toward the Kattegat. All three rivers are fed mostly by groundwater and run predominantly through agricultural land. Salmon and sea trout from these populations typically migrate into the rivers between May and November (with some individuals coming back as early as April), and spawn from late-November to early-January.

### Capture, sampling, and tagging

Between January 16 and 26, 2017, Atlantic salmon kelts (n = 66, length range 55 to 95 cm) were captured using electrofishing (Stampes Elektro, SE 500, Ringkøbing, Denmark), netted, and immediately placed in a 500 L container of fresh, oxygenated, stream water. The same process was performed to capture sea trout kelts (n = 72, length range 51 to 79 cm) between January 30 and February 11, 2017. At this period, salmon and sea trout are typically finished spawning in Denmark^31^. In addition, kelts can easily be differentiated from late spawners by their lower condition factor and the presence of scar tissue from spawning activity^32^. Within 3 minutes of capture, fish were sampled for blood (0.3 mL) at the caudal vasculature, using a 3.8-cm 25-gauge heparinized needle. Blood samples were centrifuged immediately to separate plasma from red blood cells, and placed in liquid nitrogen until further analysis. Fish were subsequently anesthetized in a solution of 0.03 g L^−1^ benzocaine in fresh water until their ventilation rate had slowed significantly. The fish were then measured (±0.5 cm), weighed (±0.01 kg) and tagged with an acoustic tag (ID-LP13, ThelmaBiotel, 28 mm length by 13 mm diameter, 9.2 g in air, 5.5 g in water) through a 2 cm incision on the left side of the fish, anterior to the pelvic fins. Each incision was closed with two sutures (4-0 vicryl absorbable sutures). The maximum tag burden was 0.84%. Scales and a fin clip were also obtained from each individual. Fish were then left to recover in a 200 L container of fresh stream water, and released at the site of capture.

A condition factor was calculated from the mass and length of each individual using the following formula (Fulton’s K):

$$Condition\,(K)=\frac{mass(g)}{{(length(cm))}^{3}}\times 100$$

Summary data of all tagged fish are presented in Table 1. Ethical note: all procedures were carried out in accordance with the regulations from the Danish Experimental Animal Committee. All experimental procedures were previously approved by the Danish Experimental Animal Committee and carried out under a license held by the Technical University of Denmark for animal experimentation (license 2017-15-0201-01164).

**Table 1:** Length, mass and condition factor (Fulton’s K) for fish of each final status.

	*Atlantic salmon*		
	Disap in river (4)	Disap in fjord (21)	Migrated to sea (41)
Length (cm)	83.9 ± 5.5	81.0 ± 5.5	79.7 ± 5.9
Mass (kg)	3.9 ± 0.94	3.8 ± 1.1	3.4 ± 0.7
Condition (K)	0.64 ± 0.06	0.69 ± 0.08	0.66 ± 0.05
	***Sea trout***		
	**Disap in river (21)**	**Disap in fjord (22)**	**Migrated to sea (29)**
Length (cm)	63.1 ± 6.3	64.9 ± 7.5	63.6 ± 8.2
Mass (kg)	2.1 ± 0.54	2.3 ± 0.91	2.2 ± 0.96
Condition (K)	0.81 ± 0.1	0.83 ± 0.07	0.83 ± 0.07

Values are mean ± standard error of the mean. Note: ‘Disap’ stands for disappeared.

### Cortisol

Cortisol was determined from blood plasma using a cortisol ELISA kit (#500360, Cayman Chemicals, Michigan, USA). This assay was previously validated for use with teleost fish plasma samples^33^. Intra- and inter-assay variations were 2.9% and 7.6%, respectively.

### Glucose

Glucose was measured from blood plasma using an Accu-Chek Mobile meter system (Roche Diabetes Care, Mannheim, Germany). This method was previously validated for use in teleost fishes^34^.

### Low molecular weight antioxidants

We used the oxygen radical absorbance capacity (ORAC) to determine overall antioxidant capacity of low molecular weight antioxidants. The ORAC assay is one of the few methods that takes the quenching reaction of reactive oxygen species (ROS) to completion, by combining both the time and percentage of ROS quenching by antioxidants, and converts it into a single quantity^35^. All analyses were performed as described in Birnie-Gauvin *et al*.^30^. Final antioxidant capacity values are reported in Trolox equivalents (TE) per μg total protein. All samples were run in duplicates (mean values were calculated and used for analysis), with an inter-assay variation of 5.61%.

### Migration timing and final status

Automatic listening stations (ALS, either Vemco VR2W, or ThelmaBiotel TBR700) were strategically deployed at the river estuary, and fjord mouth (both on the inner fjord and on the seaside), which allowed tracking of kelts as they progressed during their seaward migration. ALS were initially deployed in January 2017, and have been maintained every 5–6 weeks since. Testing of the ALS detection efficiency with both static and moving dummy tags showed 100% efficiency at all 3 river mouths, 95% at the outlet of Ringkøbing Fjord and 94% at the Limfjord outlet. Acoustic data were cleared of erroneous detections through the analysis of isolated tag detections and unrealistic fish behaviour (e.g., extreme movement speed). Fish with no detections were considered to have disappeared in the river (‘disap in river’). Fish detected at the river exit into the estuary were considered to have successfully migrated out of the river. They were considered to have disappeared in the fjord (‘disap in fjord’) if they were detected leaving the river, but not detected at the seaside of the fjord outlet into the North Sea or Kattegat. Fish detected on the seaside were characterized as having successfully migrated to sea (‘migrated to sea’). ALS detection efficiency was measured by comparing detections between consecutive arrays (e.g., number of individual fish recorded at an outward site vs. and inward site).

### Statistical analyses

Data were first investigated for outliers and collinearity. Given autocorrelation between length and mass, we used length only for further analysis. All data presented no outlier except for sea trout ORAC values. These values were log-transformed and square-root transformed in model 1 and 2, respectively. We used different transformations for each model to avoid overstretching of the predicted planes caused by the outliers. No outliers were present following the transformations.

To explore relationships between physiological parameters and migration timing (river exit), we used general linear models (GLM). We followed a step-by-step approach of model simplification from a full model (including all physiological parameters, length and condition) based on the Akaike information criterion (AIC), and tested the significance with likelihood ratio tests (LRT). Indication of overdispersion with Gaussian and Poisson distributions led us to use a GLM with negative binomial distribution and log link function. The best model fit suggested that cortisol and condition had a significant effect on day of exit in salmon (R^2^ = 0.198), and that cortisol and ORAC had a significant effect in sea trout (R^2^ = 0.597). The final models were as below:

$$\begin{array}{ll}Salmo\,salar & Salmo\,trutta\\ {\rm{Day}}\,{\rm{of}}\,{\rm{river}}\,{{\rm{exit}}}_{{\rm{i}}}\, \sim \,{\rm{NB}}({\mu }_{{\rm{i}}},{\rm{k}}) & {\rm{Day}}\,{\rm{of}}\,{\rm{river}}\,{{\rm{exit}}}_{{\rm{i}}}\, \sim \,{\rm{NB}}({\mu }_{{\rm{i}}},{\rm{k}})\\ {\rm{E}}({\rm{Day}}\,{\rm{of}}\,{\rm{river}}\,{{\rm{exit}}}_{{\rm{i}}})={{\rm{\mu }}}_{{\rm{i}}} & {\rm{E}}({\rm{Day}}\,{\rm{of}}\,{\rm{river}}\,{{\rm{exit}}}_{{\rm{i}}})={{\rm{\mu }}}_{{\rm{i}}}\\ {\rm{var}}({\rm{Day}}\,{\rm{of}}\,{\rm{river}}\,{{\rm{exit}}}_{{\rm{i}}})={{\rm{\mu }}}_{{\rm{i}}}+{{\rm{\mu }}}_{{\rm{i}}}^{2}/{\rm{k}} & \mathrm{var}(\mathrm{Day}\,{\rm{of}}\,{\rm{river}}\,{{\rm{exit}}}_{{\rm{i}}})={{\rm{\mu }}}_{{\rm{i}}}+{{\rm{\mu }}}_{{\rm{i}}}^{2}/{\rm{k}}\\ \mathrm{log}({{\rm{\mu }}}_{{\rm{i}}})={{\rm{\eta }}}_{1} & \mathrm{log}({{\rm{\mu }}}_{{\rm{i}}})={{\rm{\eta }}}_{1}\\ {{\rm{\eta }}}_{{\rm{1}}}={\rm{\alpha }}+{{\rm{\beta }}}_{{\rm{1}}}\cdot {{\rm{Cortisol}}}_{{\rm{i}}}+{{\rm{\beta }}}_{{\rm{2}}}\cdot {{\rm{Condition}}}_{{\rm{i}}} & {{\rm{\eta }}}_{1}={\rm{\alpha }}+{{\rm{\beta }}}_{1}\cdot {{\rm{Cortisol}}}_{{\rm{i}}}+{{\rm{\beta }}}_{{\rm{2}}}\cdot \,\mathrm{log}\,{({\rm{ORAC}})}_{{\rm{i}}}\end{array}$$

Where α is the intercept, and β is the slope. To explore relationships between physiological parameters and migration success (detected at sea = success), we used a general linear model with Bernoulli distribution and logit link function. The Bernoulli distribution is used when only two discrete possible outcomes are possible, in our case “success” for reaching sea, and “failure” for not doing so. Once more, we followed a step-by-step approach of model simplification from a full model (including all physiological parameters, length and condition) based on the Akaike information criterion (AIC), and tested the significance with likelihood ratio tests (LRT). The best model fit revealed that cortisol and length had a significant effect on the probability of reaching sea in salmon (R^2^ = 0.104), and that cortisol and ORAC had a significant effect in sea trout (R^2^ = 0.066). The final models were as below:

$$\begin{array}{ll}Salmo\,salar & \,Salmo\,trutta\\ {{\rm{success}}}_{{\rm{i}}}\, \sim \,{\rm{B}}(1,{{\rm{\pi }}}_{{\rm{i}}}) & {{\rm{success}}}_{{\rm{i}}}\, \sim \,{\rm{B}}({\rm{1}},{{\rm{\pi }}}_{{\rm{i}}})\\ {\rm{E}}({{\rm{success}}}_{{\rm{i}}})={{\rm{\pi }}}_{{\rm{i}}} & {\rm{E}}({{\rm{success}}}_{{\rm{i}}})={{\rm{\pi }}}_{{\rm{i}}}\\ {\rm{var}}({{\rm{success}}}_{{\rm{i}}})={{\rm{\pi }}}_{{\rm{i}}}\cdot (1\mbox{--}{{\rm{\pi }}}_{{\rm{i}}}) & {\rm{var}}({{\rm{success}}}_{{\rm{i}}})={{\rm{\pi }}}_{{\rm{i}}}\cdot (1\mbox{--}{{\rm{\pi }}}_{{\rm{i}}})\\ {\mathrm{logit}({\rm{\pi }}}_{{\rm{i}}})={{\rm{\eta }}}_{{\rm{1}}} & {\mathrm{logit}({\rm{\pi }}}_{{\rm{i}}})={{\rm{\eta }}}_{{\rm{1}}}\\ {{\rm{\eta }}}_{{\rm{1}}}={\rm{\alpha }}+{{\rm{\beta }}}_{{\rm{1}}}\cdot {{\rm{Cortisol}}}_{{\rm{i}}}+{{\rm{\beta }}}_{{\rm{2}}}\cdot {{\rm{Length}}}_{{\rm{i}}} & {{\rm{\eta }}}_{{\rm{1}}}={{\rm{\alpha }}+{\rm{\beta }}}_{{\rm{1}}}\cdot {{\rm{Cortisol}}}_{{\rm{i}}}+{{\rm{\beta }}}_{{\rm{2}}}\cdot {\mathrm{sqrt}(\mathrm{ORAC})}_{{\rm{i}}}\end{array}$$

Where success_i_ is assumed to follow a Bernoulli distribution with probability π_i_, α is the intercept and β is the slope. For model outputs, see Supplementary Table 1. All statistical analyses were performed using R version 1.1.383^36^, using lattice^37^, ggplot2^38^ and MASS^39^ R packages.

## Results

### Atlantic salmon

**Figure 2:**
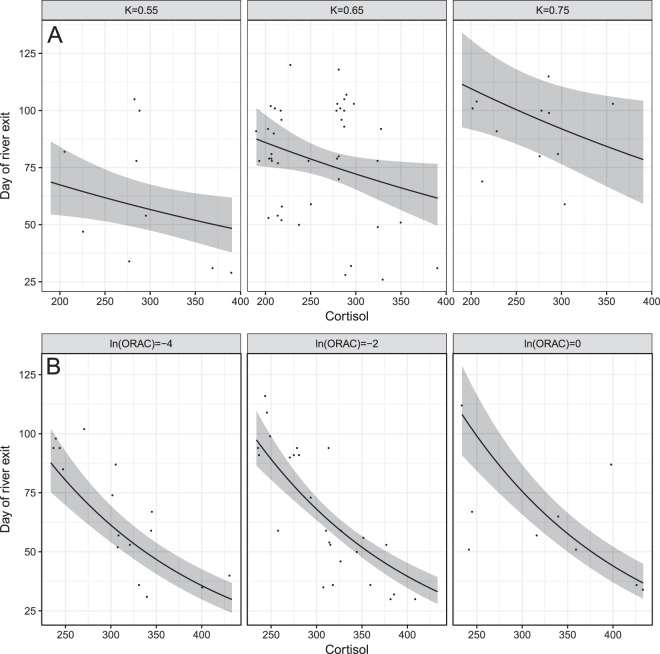
Modelled day of river exit as a function of baseline cortisol (pg per ml) levels, (**A**) at three condition factor (K) in Atlantic salmon (*Salmo salar*) kelts (*n* = 62), and (**B**) at three lnORAC in sea trout (*Salmo trutta*) kelts (*n* = 72). Models suggest a negative relationship between day of river exit and cortisol, with condition and ORAC values as important covariates for salmon and sea trout, respectively.

**Figure 3:**
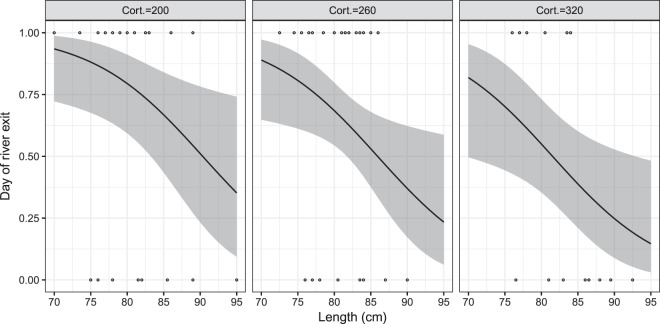
Modelled probability of reaching sea as a function of length in Atlantic salmon (*Salmo salar*) kelts at three baseline cortisol (pg per ml) measurements. Shaded area represents 95% confidence interval. Models suggest a negative relationship between probability of reaching sea and length.

**Figure 4:**
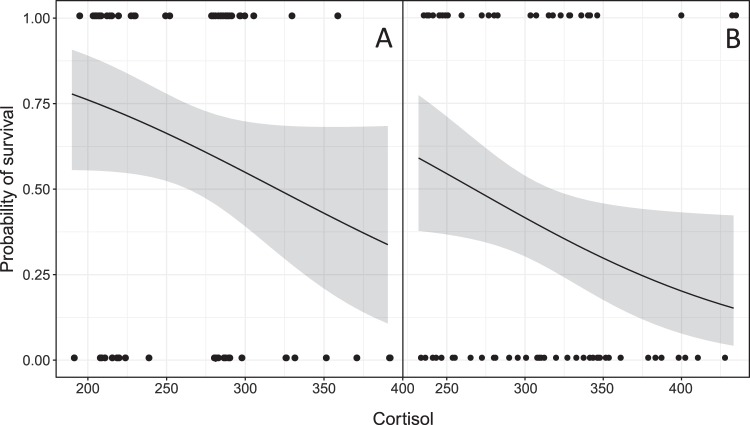
Modelled probability of reaching sea as a function of baseline cortisol (pg per ml) levels in (**A**) Atlantic salmon (*Salmo salar*) kelts assuming a length of 81.1 cm (corresponding to the mean length of tagged fish), and (**B**) sea trout (*Salmo trutta*) kelts assuming ORAC values of 0.41 (corresponding to the mean ORAC value of tagged fish). Shaded area represents 95% confidence interval. Models suggest a negative relationship between probability of reaching sea and cortisol.

In total, 41 salmon successfully migrated to sea while 21 individuals disappeared in the fjord, and 4 disappeared in the river. Cortisol (LRT: χ^2^ = 4.07, df = 1, *P* = 0.044, Fig. 2A) and condition (LRT: χ^2^ = 9.84, df = 1, *P* = 0.0017, Fig. 2A) were both significantly negatively correlated to day of river exit (beginning of migration). Length (LRT: χ^2^ = 5.75´6, df = 1, *P* = 0.016, Fig. 3) was significantly lower in individuals that successfully migrated to sea. Cortisol was also lower (nearly significantly) in individuals that successfully migrated to sea (LRT: χ^2^ = 4.0, df = 1, *P* = 0.07, Fig. 4A).

### Sea trout

In total, 29 sea trout successfully migrated to sea while 22 disappeared in the fjord, and 21 disappeared in the river. Cortisol (LRT: χ^2^ = 70.3, df = 1, *P* < 0.01, Fig. 2B) was significantly negatively correlated to day of river exit (beginning of migration). ORAC values were nearly correlated to day of river exit (LRT: χ^2^ = 3.53, df = 1, *P* = 0.060). Cortisol (LRT: χ^2^ = 4.46, df = 1, *P* = 0.034, Fig. 4B) was also significantly lower in individuals that successfully migrated to sea, though ORAC was not (LRT: χ^2^ = 3.30, df = 1, *P* = 0.087).

## Discussion

Migration is perhaps the most challenging stage of a fish’s lifecycle^40^. Nonetheless, our understanding of the drivers and underlying mechanisms of migration timing and success in fish remains sparse. This is especially true for kelts, which are amongst the least studied life stages of anadromous fish. We would further argue that kelts provide an interesting model for studying the interplay between physiology and ecology given the physiological demands that these individuals face. After spawning (where individuals may lose up to 60–70% of their energetic resources)^32^, kelts must somehow recover and return to sea successfully in order to repeat the cycle and thereby contribute to the population’s next generation. Failure to do so may affect the population greatly, though the magnitude of the effect is currently unknown. Understanding the factors that affect kelts upon their return migration may help us focus conservation and management efforts to ensure their success.

Our findings suggest that cortisol may be a good predictor of migration timing and success in kelts, such that individuals with higher levels of cortisol exit the river sooner, but are less likely to successfully reach marine environments. Cortisol has been shown to be involved in the transition from salt to freshwater, and vice versa^41,42^. Perhaps, higher cortisol levels reflect readiness to enter saltwater, and may explain why these individuals exited the river sooner. Alternatively, higher baseline cortisol levels may also be indicative of a chronic activation of the stress response, though cortisol corresponds to a single step of the stress response^23^, so caution should be taken in making this assumption. If individuals with higher baseline cortisol are in fact under chronic stress, then perhaps they exited the river in an attempt to escape the ‘stressor’ (e.g., low food availability, high predation risk, or catch-and-release practices)^43–46^. Regardless of day of river exit, individuals with higher baseline cortisol levels were less likely to attain the seaside. It is possible that fish under chronic stress conditions become resource-depleted in response to long-term activation of the stress response^22,23^. These individuals may then die before reaching the sea (e.g., through predation by seals, fisheries pressure in the fjord or from exhaustion)^47^, or may opt to reside in the fjord given the relative abundance of food in comparison to the river. The latter is unlikely in salmon because the Ringkøbing Fjord is unlikely to be suitable for mature adults, and the general consensus is that salmon kelts do not stay in estuaries/fjord habitats in Denmark (data unpublished). However, sea trout have been observed to reside in the Limfjord for some time following their migration (data unpublished). In any case, high baseline cortisol may reduce an individual’s ability to contribute to the population by (1) dying or (2) choosing an environment to overwinter (i.e., the fjord) which offers less food than the alternative (i.e., the sea).

In Atlantic salmon, condition of kelts had a strong predictive value in day of river exit such that individuals of low condition left the river earlier. Taken together with our finding that individuals with high cortisol left the river earlier, these results may suggest that early river exit is a response to stressful stimuli. Low condition may indicate low energetic state and low resource availability, which may provide a stressful stimulus, thus activating the stress response, and causing elevated baseline cortisol. Alternatively, and perhaps more likely, sustained high levels of cortisol may have derived resources from muscle mass, proteins, and lipids, and caused a decrease in condition^48^.

Cortisol increases the rate of conversion of stored energy reserves to glucose through the activation of the stress axis^23^. We may therefore expect that individuals with higher baseline cortisol would have higher baseline glucose levels. Our results indicate this was not the case, and that glucose had no predictive value in migration timing or success. Following spawning, kelts are relatively resource-depleted^32^, and may not have the energy stores available for glucose conversion, though this is unlikely, given that chronic stress will convert even muscle mass to glucose. However, glucose can also be influenced by recent feeding history which can lead to additional variation in this metric, potentially masking relationships^49^. Alternatively, it is possible that the cortisol in individuals with high baseline cortisol levels was the result of the activation of a different biochemical pathway, such as the one involved in osmoregulation for transitions between marine and freshwater environments^42^. This pathway may not result in increased rates of gluconeogenesis, and would explain the lack of correlation between glucose and cortisol levels.

We found a nearly significant association between antioxidants (ORAC values) and migration timing as well as success in sea trout, though this association was missing in Atlantic salmon. In both salmon and sea trout however, antioxidant capacity appeared low in most individuals, with some individuals having true zero values. These low capacities may reflect a depletion of antioxidant following spawning migrations, or due to a lack of resources to replenish them (e.g., low feeding for dietary antioxidant or low energy resources to generate enzymatic antioxidants). This may in fact explain why almost all individuals showed little capacity given that every individual underwent a spawning migration. Based on previous findings from juvenile brown trout, we may have expected that individuals exiting the river early would have higher antioxidant capacity as a means of preparing for migration^30^. However, because these early migrants had higher cortisol levels, and that prolonged high levels of cortisol can lead to oxidative stress^50^, it is possible that these individuals previously had higher antioxidant capacity, which was subsequently diminished following a surge in baseline cortisol, perhaps as they were preparing for their entry in saltwater.

While cortisol was identified as an important predictor for migration timing and success in both species, condition and length appeared to be important factors for salmon, while antioxidant capacity appeared to be an important factor in sea trout. The reason behind this finding is unknown, but it may be because Atlantic salmon tend to migrate significantly longer distances^1^, and energetic resources may be more critical in determining migration success to feeding areas than in sea trout, which typically migrate shorter distances. Because salmon in better condition left the river earlier, and those of shorter lengths were more likely to attain sea, it is possible that a salmon’s energetic status (i.e., condition) dictates migration initiation, but once in the fjord being shorter is somehow advantageous, perhaps in avoiding predation by seals for example^51^. Previous studies have also found that the proportion of repeat spawners decreases with length, and suggested this may be related to energy expenditure during spawning, with larger fish spending more energy, and being subsequently less likely to successfully migrate back to sea^1^. More studies should consider comparisons between Atlantic salmon and sea trout as this may allow a greater understanding of the underpinning mechanisms that drive species differences.

**Figure 5:**
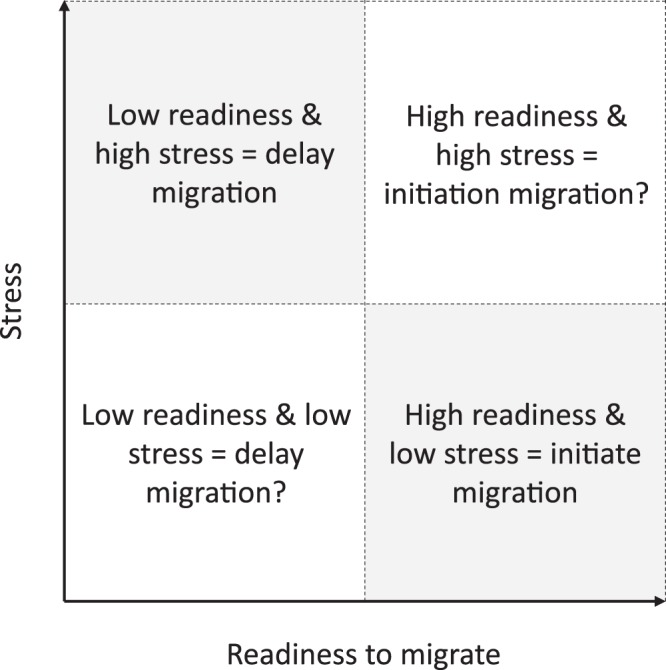
Conceptual diagram of hypothesized trade-offs in kelt migration, where individuals migrate based on a complex interplay between stress and readiness to migrate.

Taken together, these findings suggest that migration timing is a trade-off, or perhaps a complex interplay, between readiness to migrate (e.g., salt/water balance) and stressful conditions (e.g., low energetic state, environmental stimuli, predation) (Fig. 5). This study was the first that we know of to have investigated the possible link between physiological status and migration in both Atlantic salmon and sea trout kelts. We urge more studies to consider this life stage, as it represents a potentially important component of the adult population. We encourage future research on the physiological and molecular processes at play during migration, such as oxidative stress processes (e.g., DNA damage, lipid damage, telomere length), osmoregulatory processes, gene transcription (e.g., upregulation and downregulation of targeted proteins), diseases and the gut microbiome, as these may provide valuable insight in the mechanisms underlying migration patterns and variation in individual fitness.

## Supplementary information

Supplementary Table 1

## Data Availability

Data will be made accessible on figshare upon acceptance of the manuscript 10.6084/m9.figshare.7252574.v1.
