# Supplementary material for: Cortisol predicts migration timing and success in both Atlantic salmon and sea trout kelts
Source: Sci Rep. 2019 Feb 20;9:2422. doi: 10.1038/s41598-019-39153-x (PMC6382858; doi:10.1038/s41598-019-39153-x)
Supplement: Supplementary file 1 — Supplementary Table 1 [file 41598_2019_39153_MOESM1_ESM.docx]

**Supplementary Materials**

**Title: Cortisol predicts migration timing and success in both Atlantic salmon and sea trout kelts**

*In prep for Scientific Reports*

Kim Birnie-Gauvin^1^, Hugo Flávio^1^, Martin L. Kristensen^1^, Sarah Walton Rabideau^2^, Steven J. Cooke^2^, William G. Willmore^3^, Anders Koed^1^, Kim Aarestrup^1^

^1^ DTU Aqua, National Institute of Aquatic Resources, Section for Freshwater Fisheries Ecology, Technical University of Denmark, Vejlsøvej 39, 8600 Silkeborg, Denmark

^2^ Fish Ecology and Conservation Physiology Laboratory, Department of Biology, Carleton University, 1125 Colonel By Dr, Ottawa K1S 5B6, Canada

^3^ Institute of Biochemistry, Department of Biology and Chemistry, Carleton University, 1125 Colonel By Dr, Ottawa K1S 5B6, Canada

Author for correspondence: K. Birnie-Gauvin

[kbir@aqua.dtu.dk](mailto:kbir@aqua.dtu.dk)

**Running title: Physiology of outmigrating kelts**

***Supplementary Table 1*.** Model outputs. Full models include cortisol, glucose, ORAC, length and condition. Each subsequent model has one or more parameters removed. AIC = Aikake Information Criterion.

|  | **Variables removed** | **AIC** | **R^2^** |
| --- | --- | --- | --- |
| **Day of river exit** | | | |
| *Atlantic salmon* | | | |
| Full model | - | 582.39 | 0.214 |
|  | ORAC | 580.39 | 0.214 |
|  | ORAC, length | 578.56 | 0.212 |
|  | ORAC, length, glucose | 577.66 | 0.198 |
| *Sea trout* | | | |
| Full model | - | 431.80 | 0.627 |
|  | Length | 430.90 | 0.619 |
|  | Length, condition | 429.97 | 0.611 |
|  | Length, condition, glucose | 429.72 | 0.597 |
| **Probability of reaching sea** | | | |
| *Atlantic salmon* | | | |
| Full model | - | 88.29 | 0.119 |
|  | Condition | 86.37 | 0.118 |
|  | Condition, ORAC | 84.90 | 0.112 |
|  | Condition, ORAC, glucose | 83.59 | 0.104 |
| *Sea trout* | | | |
| Full model | - | 101.64 | 0.077 |
|  | Length | 99.75 | 0.075 |
|  | Length, condition | 98.05 | 0.072 |
|  | Length, condition, glucose | 96.63 | 0.066 |
